# The British Attitude Towards Vaccination Laws

**Published:** 1913-11-15

**Authors:** 


### THE BRITISH ATTITUDE TOWARDS VACCINATION LAWS.

By A CYNICAL OBSERVER.

Is it not time for the British nation to consider its vaccination laws? We should not have made the laws did not we believe that vaccination prevents infection with smallpox. Because we believe this and consider it is for the good of the individual and for the good of the community that smallpox should be prevented, we have decreed that all infants shall be vaccinated. But, although we insist on compulsory vaccination, we refuse to force vaccination on any parent who objects. This is ludicrously illogical.

Compulsion always begets resistance. No matter how salutary a law, if its purpose be to compel individuals to do something, a section of the community will actively resent it until compulsion, rigorously applied, has produced habit. Thus we now hear little of resistance to compulsory education; we hear much of resistance to compulsory insurance. If a comfortable and easy path by which to evade a law is pointed out, more and more people will take it. The ingenious resister discovered that to cry "Conscience" was the charm with which to charm the sentimental legislator. He cried "Pity me for my conscience' sake"; and by the law the conscientious objector was created. Some of the first conscientious objectors may have thought that to take this particular precaution against infection by smallpox was contrary to the will of the God in whom they believed. If they did so believe, they were justified in refusing to do that which they believed to be a sin. But most "conscientious" objectors have no conscience in the matter, and so little conscience at all, that they get up in court solemnly to swear that they object to having their children vaccinated because they believe it is contrary to the will of God, knowing full well that the real grounds of their objection are such as dislike to the trouble of a recently vaccinated baby, or a silly, sentimental objection to the infinitesimal pain of the operation.

Anti-vaccinators take advantage of the conscientious objectors' path round the law, although they do not pretend, except in court, to believe that vaccination is against God's will, therefore a sin. Out of court they make appeal to the prejudice of the ignorant by such assertions as that it is disgusting to put matter from diseased animals into the bodies of human beings. Yet they will be amongst those that clamour for the Government to supply tuberculin. Just so old maids who subscribe to the funds of the anti-vivisectionists go to dealers in animals for neuter Tom kittens.

A large percentage of our population is not vaccinated, since magistrates do not attempt the impossible task of ascertaining whether a parent has a belief or not. A little perjury and a payment of one and threepence to be permitted to perjure obtains exemption. When the public vaccinator calls parents tell him, without hesitation, that they mean to pay one and threepence for baby to be let off. It is not a wholesome state of affairs. It is absurd to say, "You must, but you need not." It is bad to force a man to act against his conscience; it is wrong to tempt him to perjury. If the community makes a law to force vaccination on every citizen, and if compliance with the law offends the conscience of any citizen, the community should not enact nor enforce a penalty against the protesting citizen, because that is legislating to force him into sin, but should take the protesting citizen and save his conscience by vaccinating him by force, or else expel him. Since it is not likely this course will be adopted, surely it is better to repeal the vaccination laws.

To make a thing difficult to get is the way to make mankind desire it. Force benefit on a man and he rejects it. Few facts are more certainly ascertained than that vaccination and re-vaccination confer immunity from smallpox. Old tradition preserves unnecessary fear of it, but actually no disease is so entirely under man's control. No one who is employed about a smallpox hospital has the fear of infection, because he knows that he is efficiently protected. None in this world need have smallpox. He who gets smallpox is in fault, because by vaccination at sufficiently frequent intervals he could have secured immunity. If in every town the community provides means for vaccination at small cost, it has done its duty by the individual. If the children of fools die, the community will not lose. If the community is not prepared to enforce a law compelling vaccination, why have any such law at all?

Whether the community keeps on the Statute-book Acts compelling vaccination or not, if the numbers of the unvaccinated much increase and severe epidemics occur, laws will be unnecessary; economic and social pressure will take their place and succeed where they have failed. Few conscientious objectors will refuse a place under Government for themselves or their children if to be confirmed in it vaccination is necessary. Such a course would entail some loss and no notoriety. So soon as employers find that they lose services by employing unvaccinated persons they will insist on vaccination as a condition of employment. The deeply-pitted daughters of the conscientious objector will find the effacement of their charms a social handicap; an obstacle to the attainment of a husband. The young man who is debarred from many employments by his conscientious objection will find his less-prejudiced rival preferred. The conscientious objector will find he can get work only with inferior firms that will ensure themselves against loss by paying low wages; that his children suffer social handicap. Not for long will he suffer ingloriously for conscience' sake. He will not find unadvertised self-martyrdom satisfying. Allowed to indulge his tender conscience secure from prosecution, he will find it grows less tender, or becomes so elsewhere.

